# Case Report: Isolated Cystic Dilatation of the Cystic Duct: A Misdiagnosed Case Preoperatively

**DOI:** 10.3389/fsurg.2021.686629

**Published:** 2021-08-10

**Authors:** Jiankang Zhang, Zeming Hu, Xuan Lin, Dongliang Zhang, Hao Wang, Bin Chen

**Affiliations:** ^1^Department of General Surgery, The First Affiliated Hospital of Gannan Medical University, Ganzhou, China; ^2^Department of General Surgery, Affiliated Xiaoshan Hospital, Hangzhou Normal University, Hangzhou, China

**Keywords:** cystic duct cyst, choledochal cyst, isolated cystic duct dilation, surgical treatment, case report

## Abstract

A 33-year-old female with a mild elevation of liver transaminase was sent to the general surgery department for medical services due to upper-right abdominal pain for 2 weeks. A liquid dark area ~4 × 3 × 3 cm in size in the theoretical location of the pancreatic segment of the common bile duct was detected by abdominal CT with no enhancement of the cystic wall found in the enhanced CT scan. The patient was then diagnosed with a choledochal cyst based on the results of the radiological images preoperatively. During the operation, the isolated cystic dilatation was found in the middle part of the cystic duct, and its caudal portion was found behind the head of the pancreas and converged into the common bile duct at an acute angle and low insertion. According to the intraoperative evaluation, the female was then diagnosed with a cystic duct cyst (CDC). The surgery was converted to a laparotomy for the unclear structure and the possibility of anatomic variation of the bile duct. The caudal portion of the cystic duct was found communicated with the common bile duct with a narrow base, and the extrahepatic bile duct was not cystic. The CDC was removed in the surgery. One week later, the patient was discharged from the hospital for the disappearance of abdominal pain and normal liver transaminase and did not report any discomfort in the 1-month-long follow-up. The lessons drawn from this case were as follows: (1) the distinction between the relatively frequent choledochal cyst and the isolated CDC should always be taken in mind; (2) a surgical strategy should be given priority for an intraoperatively confirmed CDC; (3) a common bile duct exploration is recommended for patients with choledocholithiasis or jaundice.

## Introduction

The isolated cystic dilatation of the cystic duct is a special type of bile duct cyst that is rare in the clinic and, therefore, scarcely reported in literature. The first two cases were reported by Bode et al. (1) and Serena et al. (2) consecutively in 1983 and 1991, who identified this disease as a new type of bile duct cyst, namely, a cystic duct cyst (CDC), which belongs to Todani type VI. Subsequently, some authors have proposed the subtypes VIa for the isolated CDC with narrow drainage neck to a non-dilated bile duct and VIb in other associated cysts, mainly of the main bile duct (3). The limited literature on CDCs has brought huge challenges to diagnosis in preoperative settings and therapy strategies (4). In this study, a case misdiagnosed as a choledochal cyst preoperatively instead of a CDC with the isolated cystic dilatation in the local cystic duct intraoperatively was presented. Furthermore, the available literature was reviewed to provide a reference for the clinical diagnosis and treatment of CDCs.

## Case Presentation

**Figure 1:**
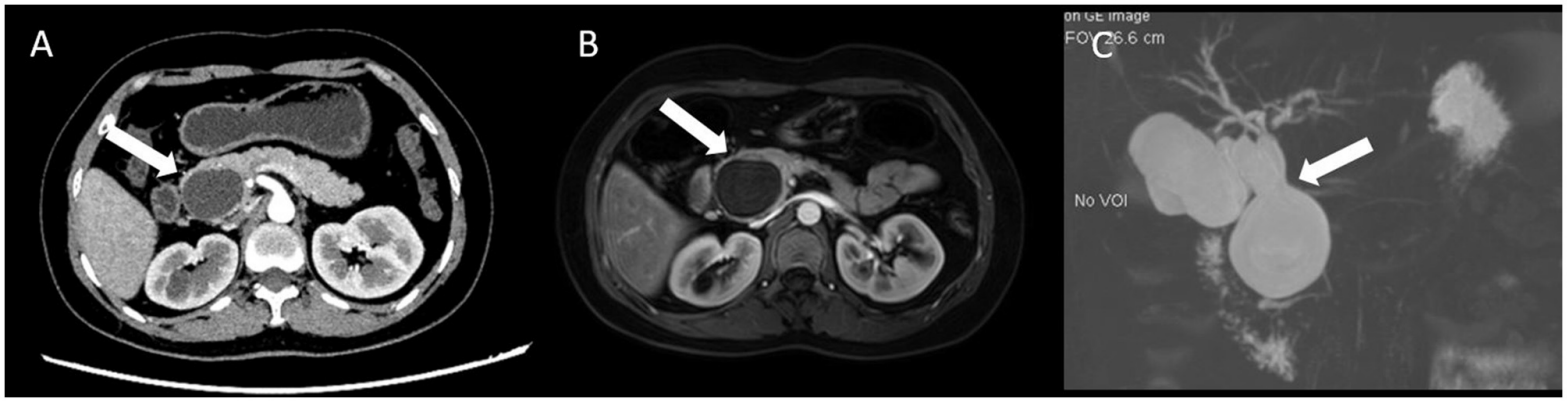
Representative abdominal CT, MRI, and MRCP images. **(A)** CT scan showed a 4 × 3 cm liquid dark area in the theoretical location of the pancreatic segment of the common bile duct, and the cystic wall showed no enhancement (white arrow); **(B)** MRI showed the spherical dilatation of the cystic dilatation (white arrow); and **(C)** Magnetic resonance cholangiopancreatography (MRCP) showed a probable choledochal cyst (white arrow).

A 33-year-old woman was sent to the general surgery department due to upper-right abdominal pain for 2 weeks. A physical examination showed that the skin color and sclera of the patient were normal; however, a mass could be felt in the upper-right abdomen. The patient had received no relevant interventions prior. Laboratory findings revealed that the liver transaminase of the patient was slightly increased: alanine aminotransferase was 120 U/L, aspartate aminotransferase was 109 U/L, and r-glutamyl transpeptidase was 180 U/L, with the levels of tumor markers and serum total bilirubin (7 μmol/L) within the normal limits. CT found a liquid dark area of about 4 × 3 × 3 cm in the theoretical location of the pancreatic segment of the common bile duct with no enhancement found in the cystic wall by the enhanced CT scan. An aspherical expansion of the cystic dilatation was detected by both MRI and magnetic resonance cholangiopancreatography (MRCP). Therefore, the patient was diagnosed with a choledochal cyst based on the results of the radiological images preoperatively (Figure 1).

**Figure 2:**
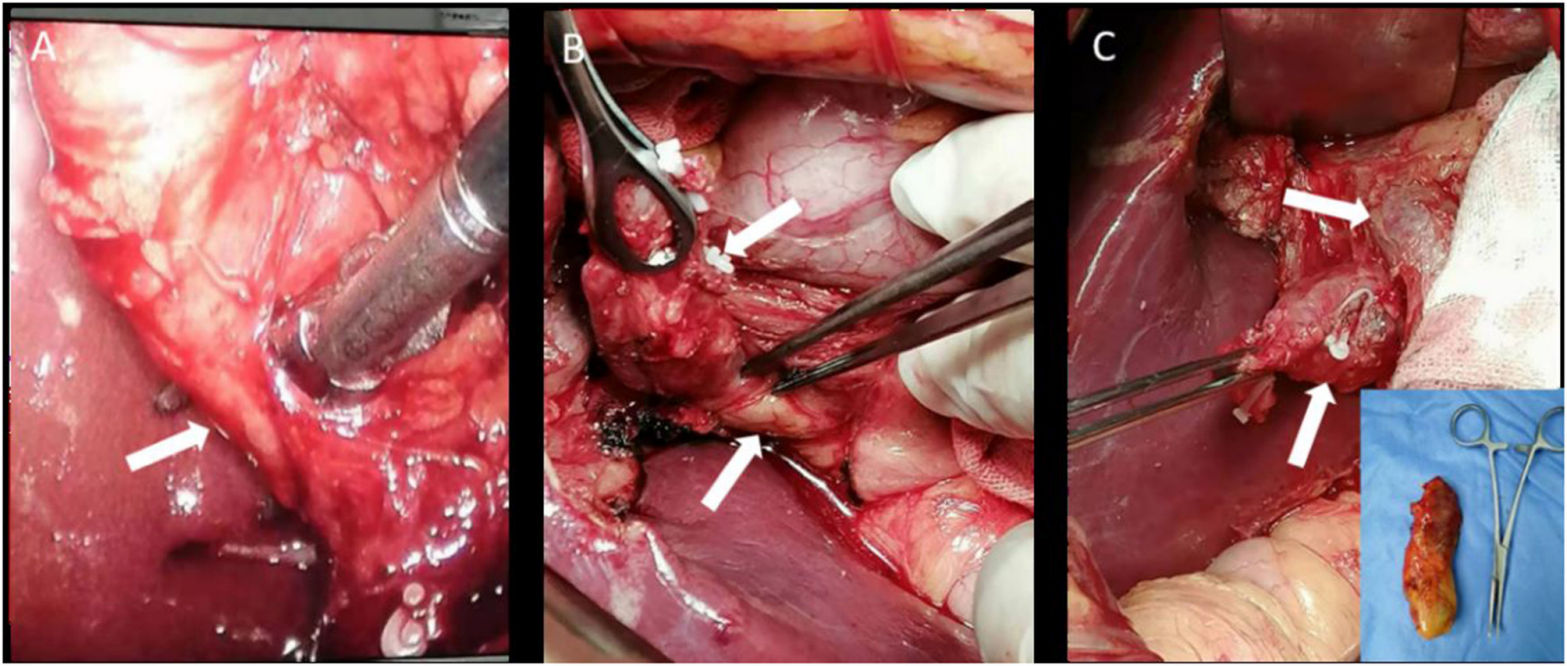
Intraoperative photographs. **(A)** Cystic duct isolation with an ultrasonic knife (white arrow); **(B)** CDC and the pancreatic head (white arrows); and **(C)** CDC and the common bile duct (white arrows). The embedded image showed the excised gallbladder specimen.

**Figure 3:**
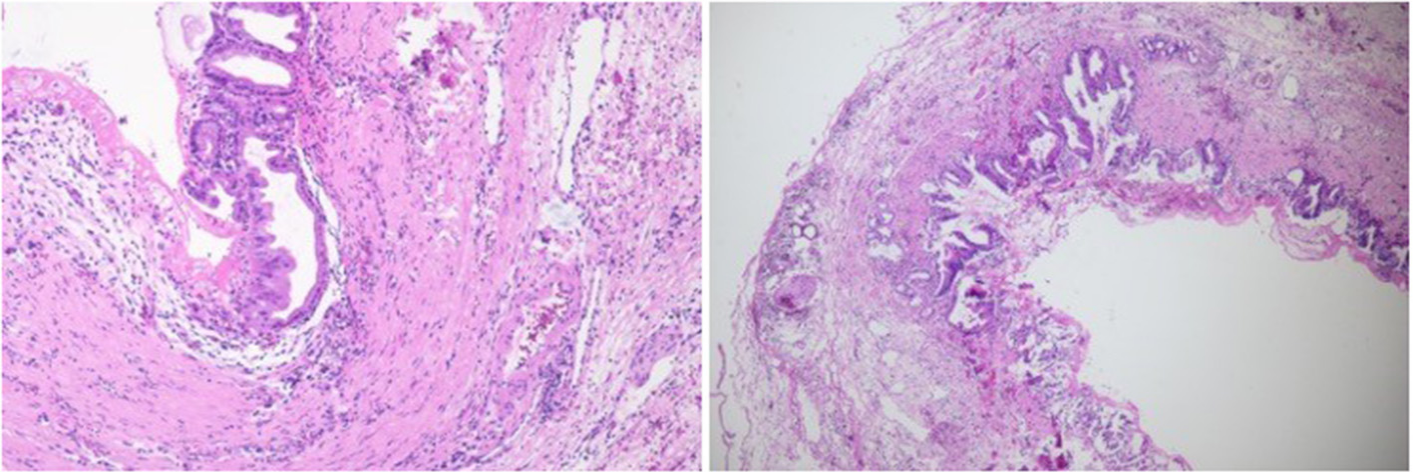
Postoperative pathological images (H&E staining). Under the microscope, a flat bile duct epithelium could be observed (×100).

Subsequently, the patient signed the informed consent and took a laparoscopic choledochal cyst resection. Under the laparoscopy, the gallbladder was found extremely dropsical with an unclear structure in the Calot's triangle. The gallbladder was resected as a routine operation, and the specimen was examined for pathology analysis. The stump of the cystic duct was lifted and separated medially along the cystic duct. The isolated cystic dilatation was found in the middle part of the cystic duct, and the caudal portion was found behind the head of the pancreas and converged into the common bile duct at an acute angle and low insertion. The surgery was converted to a laparotomy for the unclear structure and the possibility of anatomic variation of the bile duct. Because the drainage to the bile duct was through a narrow neck and the extrahepatic bile duct was not cystic, the cystic duct was eventually removed (Figure 2). Different from the preoperative diagnosis, the patient was diagnosed with a CDC based on the postoperative pathological findings (Figure 3). The abdominal pain of the patient disappeared with normal serum aminotransferase and transpeptidase. The patient was discharged a week after surgery and was asymptomatic a month later.

## Discussion

A choledochal cyst is a rare biliary malformation with an incidence of 1 in 30,000 to 1 in 50,000 in European countries with a relatively higher incidence of 1 in 1,000 in Asian countries (2, 5). The choledochal cyst is classified into five main types by Todani according to shape and location (6). However, a CDC is even rarer compared with the disease mentioned previously. Because the appearance of the cyst diverticulum is similar to that of a congenital choledochal cyst, only a few cases were misdiagnosed as type II choledochal cyst preoperatively (1, 2). There are two hypotheses about the etiology of CDC: one, in embryonic development, one side of the cystic duct wall is a congenital weakness due to the uneven development of the cystic duct wall for the lack of ganglion cells (7); two, an abnormal pancreatic-bile duct junction (APBDJ), which accounts for about 13 (2/15)−42% (21/50) of choledochal cyst cases (5, 8). In the present case, the caudal portion of the cystic duct was found behind the head of the pancreas and converged into the common bile duct at an acute angle and low insertion. Additionally, the drainage to the bile duct was through a narrow neck and the extrahepatic bile duct was not cystic, combined with the fact that pancreatic reflux would probably contribute to the development of a cystic dilatation of the main bile duct. Therefore, we think that the present case was caused by the cystic duct wall for the lack of ganglion cells.

Because the diagnosis of a CDC depends on an intraoperative exploration, the following issues should be considered preoperatively in patients with a suspected CDC: (1) patients experience typical upper-right abdominal mass and abdominal pain, but only 13 to 38% of cases have such a feature because this disease does not influence the common bile duct with no jaundice developed in the early stage (1); (2) an experienced sonographer can distinguish the cystic duct and the neck of the gallbladder by the liquid dark area (cystic dilatation); (3) the cystic structure in the head of the pancreas with a filling-defect showed by CT scan and MRI makes it difficult to distinguish the CDC from a choledochal cyst; (4) endoscopic retrograde cholangiopancreatography (ERCP) showed type I choledochal cyst; if the gallbladder is developed, the cystic duct displays a circular dilated image, the so-called dumbbell-type image.

Additionally, the postoperative pathological features were as follows: (1) the epithelium was a “simple columnar epithelium”; (2) the cystic wall had no smooth muscle cells. Combined with the fact that the caudal portion of the cystic duct was converged into the common bile duct at an acute angle and low insertion, the disease could be caused by a congenital dysplasia of the cystic duct and to a lesser extent, a long-term reverse transmission of biliary pressure. The patient showed high liver transaminases and transpeptidase levels and obvious abdominal discomfort before the operation due to the compression of the common bile duct by the cyst. Based on the pathological diagnosis of the CDC, preoperative misdiagnosis, in this case, was mainly caused by insufficient knowledge of this disease and excessive reliance on preoperative imaging examinations. Therefore, the distinction between the relatively frequent choledochal cyst and the isolated CDC should always be kept in mind.

Since CDCs are frequently accompanied by chronic cholecystitis, repeated chronic inflammation could lead to adhesion between the cyst wall and surrounding tissues, and even canceration. It was reported that early treatment of CDCs could reduce the incidence of complications (9). The surgical strategy should be given priority for the intraoperatively diagnosed CDC. There are two options for treating intraoperatively diagnosed CDC. The first is a cholecystectomy including the CDC that is precisely enough oncologically, which is recommended if the drainage to the bile duct is through a narrow neck and the extrahepatic bile duct is not cystic. The second is a cholecystectomy with excision of the extrahepatic bile duct combined with an end-to-side and hepaticojejunostomy, which is recommended for cases with a wide drainage neck or a choledochal cyst coexisting with the CDC (3, 5, 10, 11).

Moreover, common bile duct exploration should be performed simultaneously in patients with common bile duct compression, common bile duct calculi, and preoperative jaundice; if necessary, an intraoperative cholangiography (12, 13). The key to treating a CDC is to avoid damage to the common bile duct and the common hepatic duct during separation when the cyst adheres to the surrounding tissue, with the goal of reducing postoperative complications such as a bile leakage or iatrogenic bile duct injury. In the present case, the CDC was attached to the common bile duct, which was likely to be misdiagnosed under laparoscopy, and the common bile duct was subject to injure during separation. Therefore, the surgery was converted to a laparotomy for the unclear structure and the possibility of anatomic variation of the bile duct. Meanwhile, the common bile duct exploration was not performed because of the absence of choledocholithiasis and preoperative jaundice symptoms.

## Conclusion

A CDC is a rare biliary disease characterized by abdominal mass, abdominal pain, and jaundice. It is difficult to distinguish from the choledochal cyst in clinical manifestations and preoperative examinations. The present case indicated that the distinction between the choledochal cyst and the CDC should be of great importance in preoperative examinations and the adoption of appropriate surgical methods during operation to avoid postoperative complications such as a bile leakage or iatrogenic bile duct injury should be considered.

## Data Availability Statement

The original contributions presented in the study are included in the article/supplementary material, further inquiries can be directed to the corresponding author/s.

## Ethics Statement

The studies involving human participants were reviewed and approved by the First Affiliated Hospital of Gannan Medical University. The patients/participants provided their written informed consent to participate in this study. Written informed consent was obtained from the individual(s) for the publication of any potentially identifiable images or data included in this article.

## Author Contributions

JZ and ZH designed the study and drafted the report. XL, DZ, and HW prepared the figure, researched the literature, and interpreted data. BC critically reviewed the report. All authors were involved in patient management and approved the final manuscript.

## Conflict of Interest

The authors declare that the research was conducted in the absence of any commercial or financial relationships that could be construed as a potential conflict of interest.

## Publisher's Note

All claims expressed in this article are solely those of the authors and do not necessarily represent those of their affiliated organizations, or those of the publisher, the editors and the reviewers. Any product that may be evaluated in this article, or claim that may be made by its manufacturer, is not guaranteed or endorsed by the publisher.

## References

[1] Bode WE Bradley AJ. Isolated cystic dilatation of the cystic duct. J Surg Case Rep. (1983) 145:828–9. 10.1016/0002-9610(83)90152-6 6859422

[2] Serradel AS Santamaría LE Herrera GR. Cystic dilatation of the cystic duct: a new type of biliary cyst. Surgery. (1991) 109 (3 Pt 1):320–2. 2000564

[3] Perfecto Valero A Gastaca Mateo M Prieto Calvo M Ortiz de Urbina López J Valdivieso López A. Biliary cyst of the cystic duct: a case of Todani type VI. Quiste biliar del conducto cístico. Un caso de Todani tipo VI. Cir Esp. (2018) 96:659–61. 10.1016/j.ciresp.2018.04.013 30017061

[4] Miyano T Yamataka A. Choledochal cysts. Curr Opin Pediatr. (1997) 9:283–8. 10.1097/00008480-199706000-00018 9229170

[5] Kilambi R Singh A Madhusudhan K Das P Pal S. Choledochal cyst of the proximal cystic duct a taxonomical and therapeutic conundrum. Ann R Coll Surg Engl. (2018) 100:e34–7. 10.1308/rcsann.2017.0201 29181996 PMC5838701

[6] Todani T Watanabe Y Narusue M Tabuchi K Okajima K. Congenital bile duct cysts: classification, operative procedures, and review of thirty-seven cases including cancer arising from choledochal cyst. Am J Surg. (1977) 134:263–9. 10.1016/0002-9610(77)90359-2 889044

[7] Shimotake T Iwai N Yanagihara J Inoue K Fushiki S. Innervation patterns in congenital biliary dilatation. Eur J Pediatr Surg. (1995) 5:265–70. 10.1055/s-2008-1066221 8555126

[8] Bheerappa N Sastry RA. Pancreatico-biliary ductal union. Trop Gastroenterol. (2001) 22:190–3. 10.1136/gut.31.10.1144 11963322

[9] de Vries JS de Vries S Aronson DC Bosman DK Rauws EA Bosma A. Choledochal cysts: age of presentation, symptoms, and late complications related to Todani's classification. J Pediatr Surg. (2002) 37:1568–73. 10.1053/jpsu.2002.36186 12407541

[10] Perfecto Valero A Gastaca Mateo M Prieto Calvo M. Cystic duct cyst: type VI in Todani's classification. Rev Esp Enferm Dig. (2021) 113:228–9. 10.17235/reed.2020.7249/2020 33213172

[11] De U Das S Sarkar S. Type VI choledochal cyst revisited. Singapore Med J. (2011) 52:e91–3. 10.1016/j.revmed.2010.08.023 21633759

[12] Çamlidag I Nural MS Danaci M Karabiçak I Karabulut K. Cholangiocarcinoma arising from a type VI biliary cyst: a case report and review of the literature. Case Rep Radiol. (2015) 2015:625715. 10.1155/2015/625715 27034876 PMC4806668

[13] Nambiar L Alex A Siskind E Shen AW Fan C Grimaldi G. Type VI choledochal cyst-an unusual presentation of jaundice. Int J Angiol. (2016) 25:263–5. 10.1055/s-0034-1376317 27867293 PMC5114137

